# Regulating 3D Phase in Quasi‐2D Perovskite Films for High‐Performance and Stable Photodetectors

**DOI:** 10.1002/advs.202302917

**Published:** 2023-07-03

**Authors:** Haipeng Di, Wen Zeng, Bo‐Han Li, Feiyi Liao, Chen Zhao, Chuanhui Liang, Huang Li, Jia‐Cheng Wang, Da‐Bing Cheng, Zefeng Ren, Yiying Zhao

**Affiliations:** ^1^ Institute of Materials China Academy of Engineering Physics Jiangyou 621908 China; ^2^ State Key Laboratory of Molecular Reaction Dynamics Dalian Institute of Chemical Physics Chinese Academy of Sciences Dalian 116023 China; ^3^ University of Chinese Academy of Science 19A Yuquan Road Beijing 100049 China

**Keywords:** 3D perovskite phase, additive, carrier dynamics, photodetectors, quasi‐2D perovskites

## Abstract

The charge transport in quasi‐2D perovskites limits their applications despite the superior stability and optoelectronic properties. Herein, a novel strategy is proposed to enhance the charge transport by regulating 3D perovskite phase in quasi‐2D perovskite films. The carbohydrazide (CBH) as an additive is introduced into (PEA)_2_MA_3_Pb_4_I_13_ precursors, which slows down the crystallization process and improves the phase ratio and crystal quality of the 3D phase. This structure change results in a significant improvement in charge transport and extraction, leading to the device demonstrating an almost 100% internal quantum efficiency, a peak responsivity of 0.41 A W^−1^, and a detectivity of 1.31 × 10^12^ Jones at 570 nm under 0 V bias. Furthermore, the air and moisture stability of (PEA)_2_MA_3_Pb_4_I_13_ films is not deteriorated but gets significantly improved due to the better crystal quality and the passivation of defects by the residual CBH molecule. This work demonstrates a strategy for improving the charge transport properties of quasi‐2D perovskites and also sheds light on solving the stability issue of 3D perovskite films via the proper passivation or additives, which will inspire the fast development of the perovskite community.

## Graphical abstract

This work demonstrates that regulating 3D phase is an effective strategy to improve the carrier transport properties and device performance of quasi‐2D perovskites. The CBH‐additive photodetector demonstrates an internal quantum efficiency of almost 100% at 570 nm under 0 V bias with superb stability.

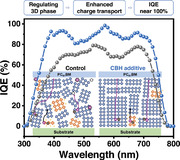

## 1 Introduction

2D perovskites have been proposed to obtain the long‐term stability via introducing organic long‐chain cations into the conventional 3D perovskite materials. However, the anisotropic carrier transport capability of 2D phases, the enhanced carrier scattering between the mixed quantum wells with multiple well widths (*n*), and potential traps formed at the interfaces of 2D phases and 2D/3D phases in solution‐processed quasi‐2D perovskite films, severely degrade the charge transport and device performance.^[^ ^1^ ^]^ The record power conversion efficiency (PCE) of quasi‐2D perovskite solar cells is 21.07%, lagging far behind that of the 3D counterparts (25.7%).^[^ ^2^ ^]^ Enhancing the charge transfer capability of quasi‐2D perovskite materials is the key to improve the device performance.

Two strategies have been proposed to enhance the carrier transport properties of quasi‐2D perovskites, aiming to minimize the above disadvantages. One approach is to avoid the anisotropic carrier transport by tuning the crystal orientation of quasi‐2D perovskite thin films to be perpendicular to the electrodes, thereby providing an efficient charge transport channel along the carrier collection direction.^[^ ^3^ ^]^ Various methods including hot‐casting deposition,^[^ ^4^ ^]^ solvent engineering,^[^ ^5^ ^]^ additive engineering,^[^ ^3^, ^6^ ^]^ solvent‐vapor annealing^[^ ^7^ ^]^ and thermal‐pressed method^[^ ^8^ ^]^ have been proposed. Tsai et al first demonstrated the hot‐casting method to improve the orientation alignment of 2D RP perovskite (BA)_2_(MA)_2_Pb_3_I_10_ and (BA)_2_(MA)_3_Pb_4_I_13_ (BA^+^ = CH_3_(CH_2_)_3_NH_3_^+^, MA^+^ = CH_3_NH_3_^+^) films.^[^ ^4a^ ^]^ Yang et al reported the synergistic effect of NH_4_Cl and H_2_O additives on rotating the crystal orientation during the (PEA)_2_(MA)_3_Pb_4_I_13_ (PEA^+^ = C_6_H_5_CH_2_NH_3_^+^) film formation, leading to high‐efficiency solar cells with a champion PCE of 17.03%.^[^ ^3^ ^]^ Zhao et al demonstrated the surface‐induced reorientation of (PEA)_2_(MA)_4_Pb_5_I_16_ films using a solvent vapor annealing method, resulting in a certified PCE of 18.00 ± 0.30%.^[^ ^7^ ^]^ Zhu et al proposed a thermal‐pressed method to prepare FPEA_2_MA_4_Pb_5_I_16_ films with uniaxial orientation, resulting in the films with single‐grain penetration along the thickness direction and photodetectors with a high responsivity of 0.4 A W^−1^.^[^ ^8^ ^]^ The above authors successfully demonstrated the effectiveness of tuning the grain orientation of 2D phases in improving the device performances.

The other strategy is to achieve the graded phases of quasi‐2D perovskite distributing along the film thickness direction and form the energy cascade energy levels to promote the carrier separation and transport.^[^ ^4^, ^5^, ^9^ ^]^ Various methods including growth condition optimization,^[^ ^4^, ^10^ ^]^ component engineering,^[^ ^11^ ^]^ and additive engineering^[^ ^4^, ^5^, ^6^, ^12^ ^]^ have been employed to regulate the phase composition distribution.^[^ ^2a^ ^]^ Hok‐Leung et al optimized the hot‐cast temperature to obtain (PEA)_2_(MA)_n−1_Pb_n_I_3n+1_ (*n* = 3 and 4) perovskite thin films with the gradient *n* phases distribution and improve the detectivity to 2 × 10^12^ Jones.^[^ ^10^ ^]^ Lai et al prepared (iBA)_2_(MA)_3_Pb_4_I_13_ films with graded phase distribution using the hot‐cast method, leading to a flexible photodetector with a high responsivity of 0.444 A W^−1^ .^[^ ^13^ ^]^ Shi et al used 4‐fluoro‐phenethylammonium (4FPEA) as an organic spacer to prepare (4FPEA)_2_(MA)_4_Pb_5_I_16_ films with vertical graded phase distribution and the corresponding solar cells displayed a high PCE of 17.3%.^[^ ^11b^ ^]^ Qing et al reported that quasi‐2D (PEA)_2_(MA)_3_Pb_4_I_13_ films prepared with 6.7 vol% dimethyl sulfoxide (DMSO) and 10 mol% MACl additives showed a graded distribution of multiple phases with type‐II band alignment, resulting in an improved PCE.^[^ ^5^ ^]^ Min et al achieved the gradient phase distribution of quasi‐2D (PEA)_2_(MA)_3_Pb_4_I_13_ thin films and improved the responsivity of photodetectors to 0.44 A W^−1^ and the detectivity to 3.38 × 10^12^ Jones, by adding a small amount of DMSO into the precursor.^[^ ^4b^ ^]^ The above work successfully has demonstrated that the vertical graded phase distribution can improve the device performance.

However, researchers fail to notice the fact that the 2D (small‐*n*) phases are distributed in the matrix of the 3D (*n* = ∞) phase in quasi‐2D perovskite thin films, and the carrier primarily transports in the 3D phase.^[^ ^14^ ^]^ Herein, we propose that the carrier transport properties and the device performances can be improved via regulating the 3D phase in quasi‐2D perovskite films. Carbohydrazide (CBH) is employed as an additive in the precursor to regulate the 3D phase in the quasi‐2D (PEA)_2_MA_3_Pb_4_I_13_ perovskite thin films. High‐resolution transmission electron microscopy (HRTEM) images show that the crystallinity and alignment of 3D phase are significantly improved, and the phase ratio of 3D perovskite is increased. The enhanced charge transport and extraction were revealed by the sensitive transient absorption (TA) in the linear response range (the non‐carrier density dependent region in other words) under low carrier density. The optimum devices demonstrate an IQE of 98%, a peak responsivity of 0.41 A W^−1^, and a detectivity of 1.31 × 10^12^ Jones at 570 nm under 0 V bias. Furthermore, the decomposition mechanism and the stability of (PEA)_2_MA_3_Pb_4_I_13_ thin films were systematically investigated.

## 2 Results and Discussion

**Figure 1:**
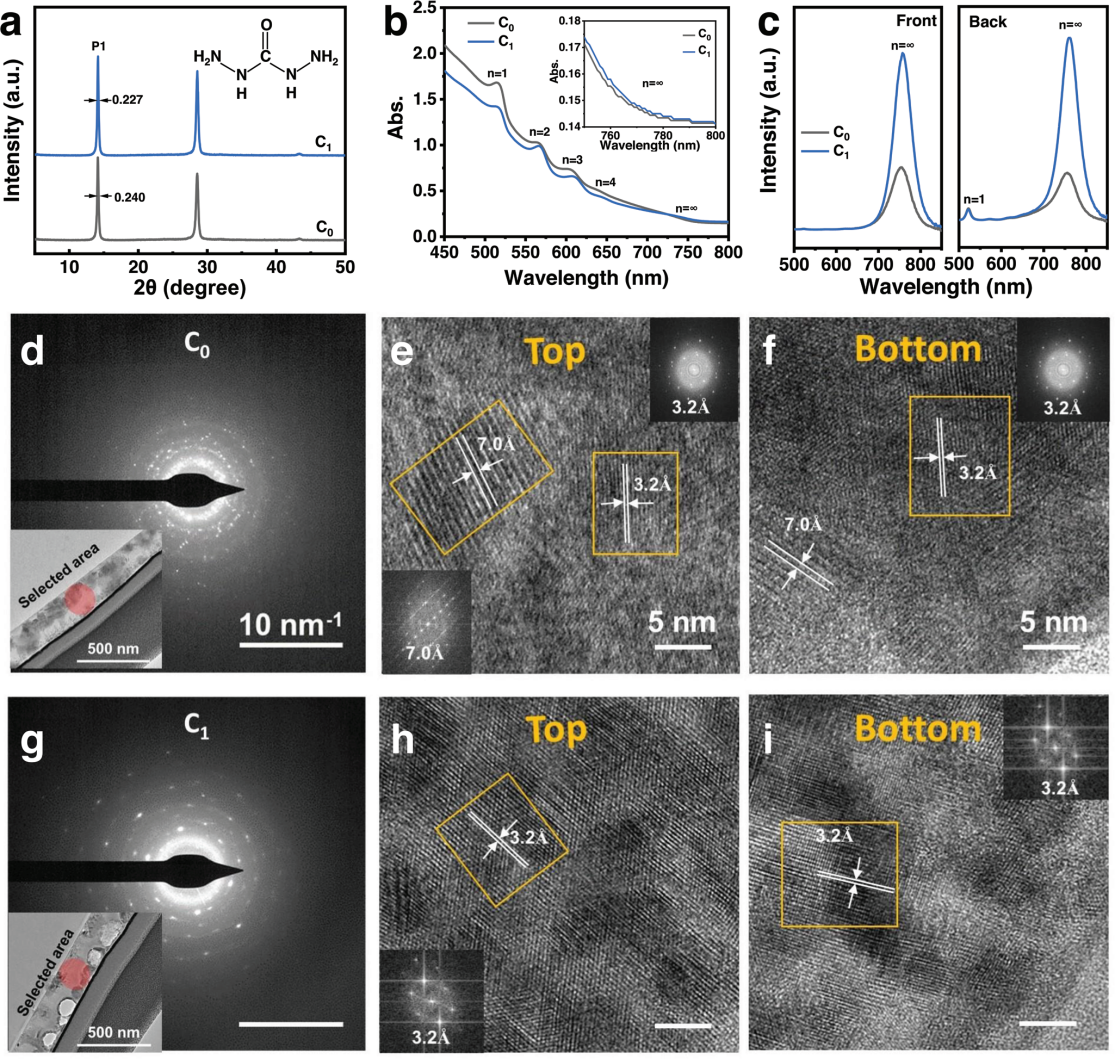
a) XRD patterns, b) UV–vis absorption spectra in transmission mode, inset: absorption spectra in wavelength of the *n* = ∞ phase of the C_0_ and C_1_ films. c) Steady‐state PL spectra of the C_0_ and C_1_ films excited with 470 nm light from the front side (the perovskite films side) and back side (the glass side). Selected‐area electron diffraction (SAED) patterns of (d) the C_0_ and g) the C_1_ films, inset: illustration of the corresponding selected region. HRTEM images and fast transform patterns of (e,f) the C_0_ and (h,i) the C_1_ films in the top and bottom regions, respectively. C_0_ and C_1_ represent the films hot‐casted with precursors without CBH and with 1% CBH molar ratio, respectively.

We chose the CBH (chemical structure shown as the inset of **Figure** **1**a) as an additive to regulate the 3D phase in the quasi‐2D perovskite (PEA)_2_MA_3_Pb_4_I_13_ films. The C=O group in CBH molecule can interact with the unfilled p‐orbitals of Pb^2+^ ,^[^ ^15^ ^]^ evidenced by a downshift of the characteristic peaks of Pb 4f_5/2_ and Pb 4f_7/2_ core level in the XPS spectra of the (PEA)_2_MA_3_Pb_4_I_13_ film with and without CBH additive in Figure S1a (Supporting Information). The amino group in CBH molecule can interact with the I^−^ sites via hydrogen bonding,^[^ ^15^, ^16^ ^]^ featured in a downshift in the core level of I 3d in the XPS spectra of the (PEA)_2_MA_3_Pb_4_I_13_ film with and without CBH additive shown in Figure S1b (Supporting Information). Therefore, it is expected that these interactions can slow down the crystallization process of perovskite films and favor the formation of the 3D perovskite phase.

The quasi‐2D perovskite films were prepared via hot‐casting using precursors with CBH molar ratio of 0, 0.5%, 1%, 1.5%, and 2%. These films were denoted as C_0_, C_0.5_, C_1_, C_1.5_, and C_2,_ respectively. The slow discoloration of the C_1_ samples in Figure S2 (Supporting Information) confirmed our expectation on the slow crystallization process caused by the CBH additive. The CBH concentration has little effect on the film thickness and morphology. As shown in Figure S3 (Supporting Information), all the films exhibit uniform, full coverage, and smooth morphologies, with an average surface roughness (Sa) of 19 nm. A few cracks can be found in the top‐view scanning electron microscopy (SEM) images (Figure S3b, Supporting Information), which are often observed in 2D perovskite films.^[^ ^14b^ ^]^ All the films exhibit a similar thickness ≈300 nm, as shown in the cross‐sectional SEM images (Figure S3c, Supporting Information). X‐ray diffraction (XRD) patterns of both C_0_ and C_1_ film in Figure 1a demonstrate three diffraction peaks located around 14.2^o^, 28.5^o^, and 43.4^o^, which correspond to the (111), (202), and (313) crystal planes of layered perovskite (PEA)_2_MA_3_Pb_4_I_13_ phase or the (110), (220), and (313) crystal planes of 3D perovskite MAPbI_3_, respectively.^[^ ^14b^ ^]^ The intensities of three diffraction peaks of the C_1_ film are increased and the full‐width at half‐maximum (FWHM) of the diffraction peak P1 at 14.2^o^ is reduced from 0.240 to 0.227 compared with the C_0_ film, indicating the improved crystallinity from the CBH additives.

The UV–vis absorption spectra in transmission mode (Figure 1b) of the C_0_ and C_1_ films show absorption peaks at ≈514, 568, 609, 645, and 750 nm, which are assigned to (PEA)_2_MA_n‐1_Pb_n_I_3n+1_ of *n* = 1, 2, 3, 4, and ∞ phase,^[^ ^4b^ ^]^ respectively. It can be concluded that those films are composed of mixed 2D phases and 3D phase. Compared with the C_0_ film, the absorption in the small‐*n* wavelength range decreases, and the absorption in the *n* = ∞ wavelength range increases in the C_1_ films, which is attributed to the higher phase ratio of *n* = ∞ phases. Steady‐state PL spectra of the C_0_ and C_1_ films in Figure 1c under the front‐ and back‐excitation at 470 nm show an emission peak at 754 nm, which can be identified as the emission of the *n* = ∞ phase.^[^ ^4b^ ^]^ The increased intensity of the emission peaks in the C_1_ film suggests again that the higher ratio and/or better quality of 3D perovskite phase are formed in quasi‐2D perovskite (PEA)_2_MA_3_Pb_4_I_13_ films due to the introduction of CBH.

HRTEM images and the selected area electron diffraction (SAED) were obtained to investigate the effect of CBH on the phase distribution and alignment. The selected area with a diameter ≈300 nm covers almost the whole cross‐section of the thin film (Figure S4a,b, Supporting Information) and is marked as a circle in Figure 1d,g. The SAED patterns of the C_0_ film in Figure 1d show three diffraction rings, indicating its polycrystalline nature. While the SAED patterns of the C_1_ film in Figure 1g show both sharp diffraction spots and the diffraction rings of the high‐index crystal planes, indicating the preferred crystal alignment and the improved crystal quality. As shown in the HRTEM images in Figure 1e,f, crystal lattices with a spacing of 3.2 and 7.0 Å, corresponding to the 3D MAPbI_3_ phase and the 2D phase,^[^ ^4b^ ^]^ respectively, are observed in both the top and bottom regions in the C_0_ film. HRTEM images in Figure 1h,i exhibit large area of clear, homogenous, and highly‐aligned 3D perovskite lattice stripes in both the top and bottom regions of the C_1_ film. These observations further confirm that the film crystallinity in the C_1_ films is significantly improved, and the 3D perovskite crystal grains are well‐aligned in the similar orientation. Less 2D phases can be found in the HRTEM images in Figure 1h,i, suggesting the suppression of formation of 2D phases in the C_1_ films. Furthermore, the HRTEM images in Figure S4c–f (Supporting Information) further demonstrate that the 3D phase grains in the C_1_ film have the larger grain sizes, the better crystallinity, and the improved crystal alignment. In conclusion, the introduction of CBH in quasi‐2D (PEA)_2_MA_3_Pb_3_I_13_ thin film can favor the growth of the 3D phase in terms of improving the crystallinity, the grain size, and the grain alignment.

**Figure 2:**
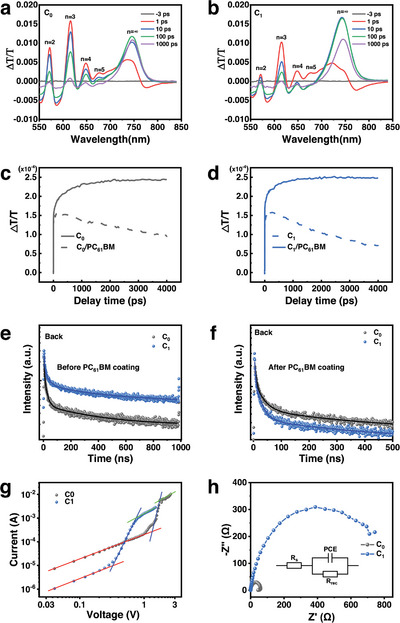
Transient absorption (TA) spectra at indicated times of a) the C_0_ and b) the C_1_ perovskite thin films under back‐excitation, where the pump energy was 100 nJ per pulse with a light spot diameter of 3 mm and wavelength of 517.5 nm. TA dynamics of the c) C_0_ and d) C_1_ films probed at the *n* *= ∞* bands under back‐excitation before PC_61_BM coating and after PC_61_BM coating, respectively. Time‐resolved photoluminescence (TRPL) spectra of the C_0_ and the C_1_ films excited by a laser of 375 nm under the back side e) before PC_61_BM coating and f) after PC_61_BM coating. g) SCLC curves of the C_0_ and the C_1_ devices. h) Typical Nyquist plots of the C_0_ and the C_1_ devices.

A sensitive TA spectrometer^[^ ^17^ ^]^ was employed to investigate the carrier dynamics of C_0_ and C_1_ films. The TA curves under back‐excitation at 517.5 nm are shown in **Figure** **2**a,b. The bleach recoveries of the *n* = 2, 3, 4, and 5 phases accompany with the bleach formation of the *n* = ∞ phase at the primary hundred picosecond, suggesting that the electrons transfer from the small‐*n* to *n* = ∞ phases within 1 nanosecond and the carrier transport mainly occurs in the 3D (*n* = ∞) phase.^[^ ^14^ ^]^ Our previous work^[^ ^18^ ^]^ have demonstrated that the TA dynamics curves in linear response range (the non‐carrier density‐dependent region) under the low carrier density reflects the genuine carrier behaviors (Figure S5, Supporting Information), so the following discussions mainly focus on the carrier dynamics of the *n* = ∞ phase in the linear response range.

The carrier dynamic curves of the *n* = ∞ phase in the C_0_ and C_1_ films under back‐excitation before and after coating the PC_61_BM layers are shown in Figure 2c,d, respectively. Before the PC_61_BM coating, after excitation, the bleaching signal of the *n* = ∞ phase rises and reaches a saturation after ≈1 ns, indicating that electrons transfer from the small‐*n* phases to the *n* = ∞ phase. The transfer rate (k*_T_*) of electrons from the small‐*n* phases to the *n* = ∞ phase is determined to be 4.56 × 10^−3^ and 5.74 × 10^−3^ ps^−1^ for the C_0_ and C_1_ film, respectively, indicating that the electron transfers faster in the C_1_ film. Detailed fitting section is shown in Table S1 and Figure S6a (Supporting Information). After the PC_61_BM coating, the bleaching signal of the *n* = ∞ phase rises first and starts to decay at ≈300 ps, due to the electron extraction of the PC_61_BM layer. The reduction of the bleaching intensity at ≈300 ps of the *n* = ∞ phase in the C_1_ film after the PC_61_BM coating in Figure 2d are obviously larger than that in the C_0_ film (Figure 2c). The extraction rates of electrons are 1.05 × 10^−4^ and 2.05 × 10^−4^ ps^−1^ at the C_0_/PC_61_BM interface and C_1_/PC_61_BM interface, respectively, as shown in Figure S6b (Supporting Information), indicating the more efficient electron extraction at the C_1_ films/PC_61_BM interface.

Time‐resolved photoluminescence (TRPL) spectra in Figure 2e and the detailed fitting parameters in Table S2 (Supporting Information) show that the average lifetime (*τ*_ave_) of perovskite films increases from 288.2 to 343.4 ns with the introduction of CBH additive. While after the PC_61_BM coating, the *τ*_ave_ of perovskite films without and with CBH additives are 263.7 and 208.2 ns, as shown in Figure 2f, respectively. This is consistent with the TA results. Hence, it can be concluded that the introduction of CBH can effectively improve the electron transport in (PEA)_2_(MA)_3_Pb_4_I_13_ thin films and the electron extraction efficiency at the perovskite film/PC_61_BM interface. Combined with the HRTEM images, the improved carrier transport and extraction can be attributed to the higher 3D perovskite phase ratio, the larger grain size, the better crystallinity, and the well‐aligned crystal orientation of the (PEA)_2_(MA)_3_Pb_4_I_13_ thin films.

The space charge limited current (SCLC) and the electrochemical impedance spectroscopy (EIS) measurements were performed to investigate the difference of the trap density (*N*_d_) and the recombination resistance (*R*_rec_) in the C_0_ and C_1_ perovskite films. As shown in Figure 2g, the trap density is largely reduced from 1.02 × 10^16^ to 3.59 × 10^15^ cm^−3^ with the introduction of CBH. The reduction of the defect density can be contributed to the improved quality of 3D phase and the passivation of defects such as *V*_I_^−^ and *V*_Pb_^2+^ in these films due to the interactions between the remained CBH additives^[^ ^19^ ^]^ and the perovskite.^[^ ^20^ ^]^ The *R*_rec_ can be obtained from the EIS spectra under dark condition by fitting the equivalent circuit model and the Nyquist plots of these devices are shown in Figure 2h. The C_1_ device shows the higher *R*_rec_ than that of the C_0_ devices, indicating the well‐suppressed carrier nonradiative recombination in the C_1_ device. Therefore, the reduction of the defect density and the nonradiative recombination can be attributed to the passivation effect of CBH and the improvement of the crystal quality of 3D phase.

**Figure 3:**
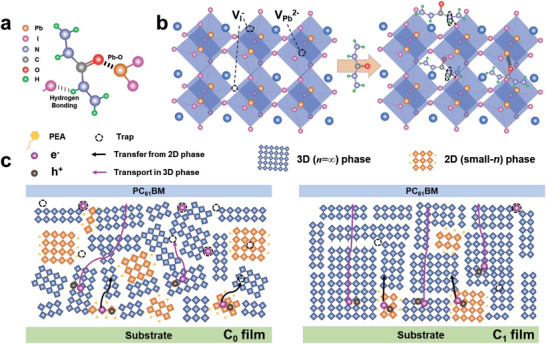
Schematic illustration of a) interactions between CBH and perovskite precursor; b) defect passivation mechanism between CBH and perovskite crystal; c) transport and extraction process of photogenerated carriers in the C_0_ and the C_1_ films.

The mechanism of how 3D perovskite phase affects the charge transport properties of quasi‐2D (PEA)_2_MA_3_Pb_4_I_13_ perovskite is schematically illustrated in **Figure** **3**a–c. The C=O group and —NH— group in CBH molecule can interact with perovskite to form lead‐coordinating bonding and hydrogen bonding as shown in Figure 3a, respectively. These interactions between CBH molecule and perovskites can greatly slow down the nucleation of perovskite from the precursor solution, leading to a slower crystallization rate. These interactions can also obstacle the diffusion of the organic cations (PEA^+^) and thereby suppress the formation of small‑*n* phases,^[^ ^21^ ^]^ resulting in a lower ratio of 2D perovskite phases and an improved crystal quality, as well as the alignment of the 3D perovskite phase, as shown in Figure 1. The decreased proportion of 2D phases can diminish the carriers scattering, and the lower density of grain boundaries and defects can reduce the non‐radiative recombination of carriers. The improvement in the crystal alignment of 3D phase also favors the carrier transport in quasi‐2D perovskite films. The residue CBH molecule within the thin film (confirmed by the FTIR absorption spectra in Figure S7a, Supporting Information and the N1s characteristic peaks^[^ ^19^ ^]^ in XPS spectra in Figure S7b, Supporting Information) can passivate the defects such as *V*_I_^−^ and *V*_Pb_^2+^ in the surface and the bulk, as illustrated in Figure 3b, leading to a lower defect density in the perovskite film. The lower defect density in the bulk and at the surface of quasi‐2D perovskite film can reduce the recombination rate of carriers and enhance the carrier extraction at the perovskite/PC_61_BM interface. All the above mechanisms work together and improve the transport properties of photogenerated carriers, as illustrated in Figure 3c. The changes in 3D phase of the quasi‐2D perovskite films including the higher phase ratio, the reduced density of defects, the enlarged grain size, and the well‐aligned crystal grains, leading to the greatly enhanced charge transport and extraction capability.

**Figure 4:**
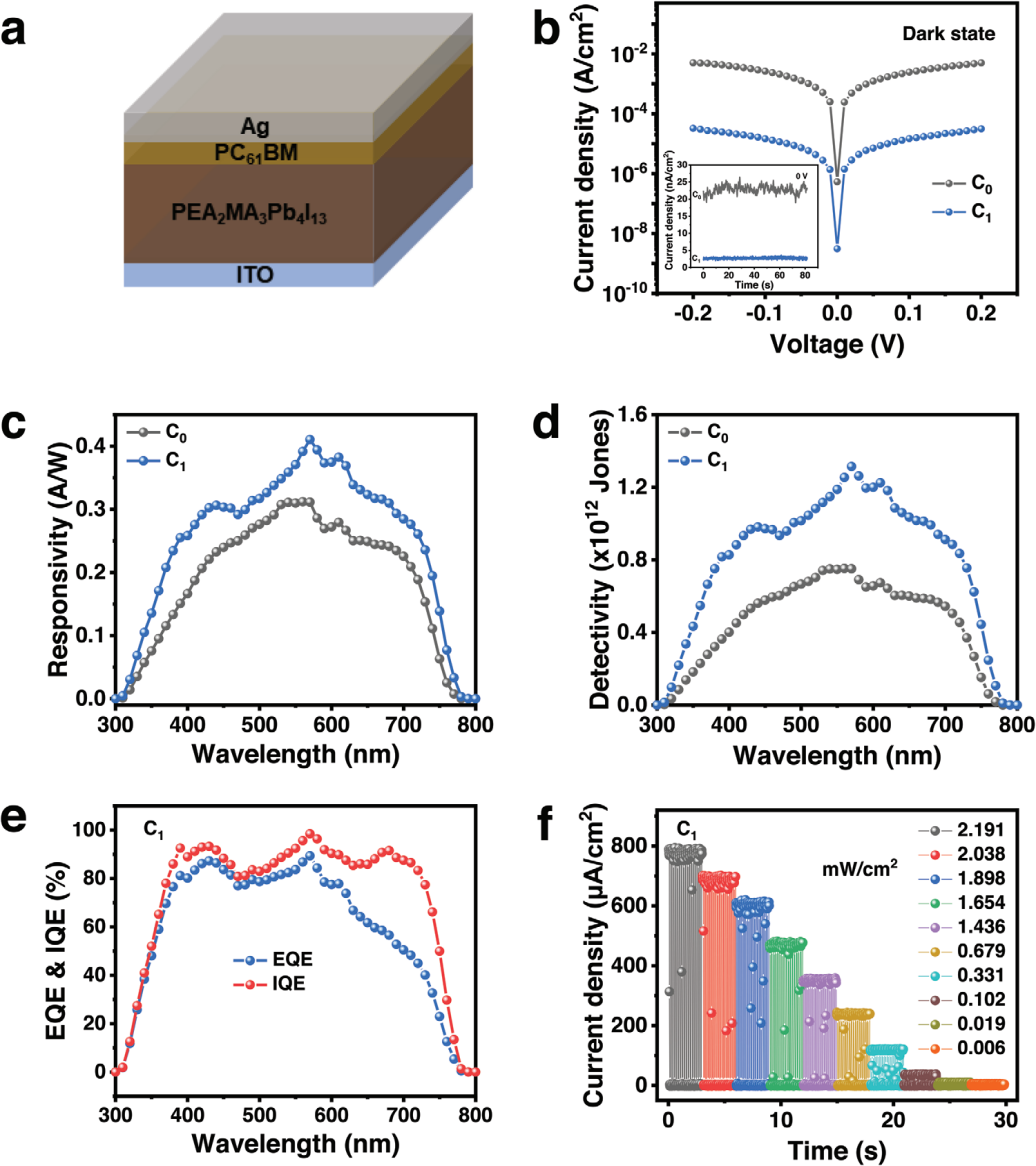
a) Schematic diagram of the device structure. b) Dark *I–V* curves and the time‐dependent *I–t* curves under 0 V bias of the C_0_ and C_1_ devices. c) Responsivity and d) detectivity at 10 Hz of the C_0_ and C_1_ devices in the wavelength ranging from 300 to 800 nm under 0 V bias. e) EQE and IQE of the C_1_ device. f) On‐off photo‐response curves of the C_1_ device under different light intensity.

Devices with the configuration of indium tin oxide (ITO)/quasi‐2D perovskites/PC_61_BM/Ag, as shown in **Figure** **4**a, were fabricated to demonstrate the effectiveness of our strategy. The dark current‐voltage (*I–V*) curves and the time‐dependent dark current (*I–T*) curves under 0 V bias in Figure 4b shows that the addition of CBH can effectively reduce the dark current of the photodetectors almost one order of magnitude due to the passivation of defects such as *V*_I_^−^ and *V*_Pb_^2+^.^[^ ^22^ ^]^ The noise current in Figure S8 (Supporting Information) is in accordance with their dark current in variation trend under 0 V bias. The responsivity of these devices as a function of wavelength under 0 V bias are plotted in Figure 4c. With the addition of CBH, the responsivity of the photodetectors is improved in the whole wavelength range of 300–800 nm, and the maximum responsivity is increased from 0.31 to 0.41 A W^−1^ at 570 nm. The detectivity is also increased in the wavelength range of 300–800 nm and the peak detectivity at 570 nm is improved from the 0.75 × 10^12^ Jones of the C_0_ device to the 1.31 × 10^12^ Jones of the C_1_ device. These parameters of photodetector are comparable to the best reported values in Table S3 (Supporting Information). The C_1_ device exhibits a higher quantum efficiency than that of the C_0_ device in general, as shown in Figure 4e and Figure S9a (Supporting Information). It is worthy to mention that the internal quantum efficiency (IQE) of the C_1_ device is almost 100% under 0 V. The drop of the external quantum efficiency (EQE) in the long wavelength region is atrributed to the reduced light absorption (Figure S9 b,c, Supporting Information). The on‐off characteristics of the C_1_ device were measured under different incident intensity. As shown in Figure 4f, the C_1_ device exhibits the stable photocurrent and no drift in the dark current under a wide range of the light intensity from 0.006 to 2.191 mW cm^−2^. The rise time (the time of photocurrent rising from 10% to 90%) and fall time (the time of photocurrent decresing from 90% to 10%) of the C_1_ device are 0.532 and 0.521 ms (Figure S9d, Supporting Information), respectively. The improvement of the IQE and EQE can be directly contributed to the enhanced electron transport and extraction, which results from the good quality and the well‐aligned grains of 3D phase in quasi‐2D perovskite thin films.

**Figure 5:**
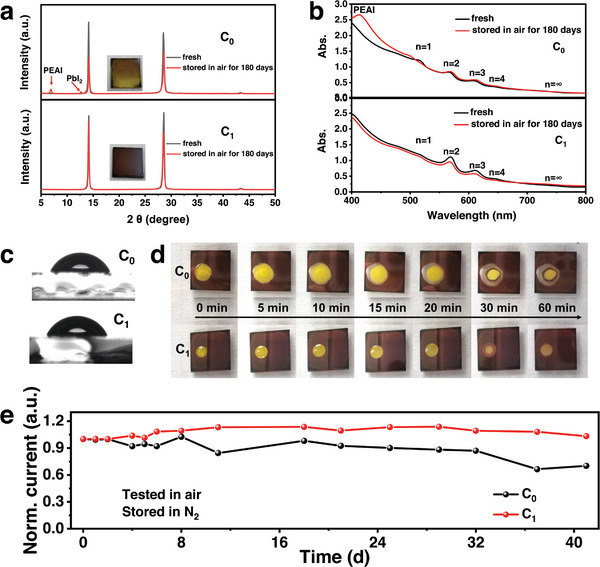
a) XRD patterns and b) UV–vis absorption spectra of the C_0_ and C_1_ films exposed in ambient air with RH of 20–30% at RT. c) Contact angles of deionized water on the C_0_ and C_1_ thin films measured at the beginning of 10s. d) Optical images of the C_0_ and C_1_ films treated with 10 µL deionized water taken at different time intervals. e) Normalized current variation curves of the devices based on the C_0_ and C_1_ thin films.

The long‐term stability of the films was also investigated to clarify whether the higher 3D perovskite phase ratio would deteriorate the stability of quasi‐2D perovskite films. **Figure** **5**a shows the XRD patterns of the C_0_ and C_1_ films stored in air with a relative humidity (RH) of 20–30% at room temperature (RT). After 180 days, the color of the C_0_ film has changed from black to light yellow and the XRD patterns of the C_0_ film exhibited two new diffraction peaks locating at 6.9^o^ and 12.6^o^, which can be assigned to diffraction of PEAI (Figure S10, Supporting Information) and PbI_2_,^[^ ^23^ ^]^ respectively. The UV–vis absorption spectrum of the C_0_ film in Figure 5b exhibits a new peak at 420 nm, which is corresponding to the absorption peak of PEAI. It can be concluded that the decomposition of the C_0_ films in air is mainly due to volatilization of the MA component and oxidation of I^−^ ions. Compared with the C_0_ film, the C_1_ film remains the dark color after 180 days and there are no extra diffraction peaks appeared in the XRD patterns (Figure 5a) and no extra absorption peaks in the UV–vis absorption spectrum (Figure 5b). The addition of CBH can effectively suppress the volatilization of the MA component, which can be attributed to the hydrogen bonding between C=O and MA (CH_3_NH_3_).^[^ ^15^, ^16^ ^]^ Similar phenomenon can also be observed in Cl—P=O system.^[^ ^21^ ^]^ Meanwhile, CBH is an reductant^[^ ^19^ ^]^ thus can effectively suppress the oxidation of I^−^ ions. Therefore, the addition of CBH can effectively suppress the decomposition of the perovskites and improve the environmental stability of the (PEA)_2_MA_3_Pb_4_I_13_ perovskite films.

The contact angles of deionized water on both films were very close (69.4^o^ and 72.8° for the C_0_ and C_1_ films) at the beginning 10 s as shown in Figure 5c. The deionized water on the C_0_ film continued to spread on the surface and began to degrade the perovskite, while the deionized water on the C_1_ film did not spread out on the surface and disappeared one hour later until the fully volatilization (Figure 5d). The improved moisture stability can be attributed to the increased surface hydrophobicity, resulting from the hydrogen bonding between —NH— group of CBH molecule and on perovskite surface.^[^ ^8^, ^15^, ^16^ ^]^ The normalized photocurrents of the C_0_ device dropped ≈30% after 41 days, while that of the C_1_ devices exhibited negligible decay. The C_1_ devices also exhibited improved stability under light illumination with an electrical bias in Figure S11 (Supporting Information). The improved device stability can be attributed to the improved air and moisture stability caused by the improved crystal quality and the passivation effect of residual CBH molecule.

We have demonstrated the improved charge transport property and stability by increasing the phase ratio of 3D phase in (PEA)_2_MA_3_Pb_4_I_13_ quasi‐2D perovskite films. The enhanced charge transport is as expected since the 3D phase has better charge transport properties than the 2D phases and the charge transport occurs in the 3D phase during most of the time. Meanwhile, it is out of our expectation that the film stability was not deteriorated but significantly improved with the increasing of the ratio of 3D phase. It is contrary to the fact that 2D phases have better stability than the 3D phase. The improved stability can be attributed to the improved crystal quality of 3D phase and the extra passivation effect from the CBH residue. This result also suggests that the 2D perovskite phases play much smaller role than expected in improving the stability of quasi‐2D perovskite films. Most importantly, it reveals a possibility that the stability of 3D films with right additives or proper passivation can be better than the 2D perovskite films. It is worthy to further investigate the passivation mechanism and additives to improve the stability of 3D perovskites, which will greatly accelerate the progress and the commercialization of the perovskites and related devices.

## 3 Conclusion

This work demonstrates that the charge transport properties and stability of quasi‐2D perovskite films can be improved through the regulation of the 3D phase by introducing a small amount of carbohydrazide (CBH) additive in the precursors. The mechanism involves the interaction between the C=O group (electron pair donor) in the CBH molecule and the unfilled p‐orbitals of Pb^2+^, forming lead‐coordinating bonding, while the amino group (—NH—) interacts with the I^−^ sites. Such interactions can effectively slow down the rapid crystallization process during the hot‐casting process and promote the alignment and crystallization of the 3D phase. Consequently, the charge transport in the perovskite film and the charge extraction efficiency at the perovskite/PCBM interface are significantly enhanced. Additionally, the residual CBH molecule can effectively passivate the surface and bulk defects of the (PEA)_2_MA_3_Pb_4_I_13_ film, leading to a lower carrier recombination rate. As a result, the optimum device shows a peak IQE of 98%, a responsivity of 0.41 A W^−1^ and a detectivity of 1.31 × 10^12^ Jones at 570 nm under 0 V bias.

Furthermore, the residual CBH molecule effectively suppresses the volatilization of the MAI component and enhances the surface hydrophobicity, leading to significantly improved environmental stability of (PEA)_2_MA_3_Pb_4_I_13_ perovskite films. The resulting devices exhibit negligible performance decay after ≈1000‐hour storage. The strategy proposed here provides insights on the charge transport and inspires the further improvement of the quasi‐2D perovskites. This work also sheds light on the stability issue and accelerates the commercialization of 3D perovskites, which could be solved by adapting the proper additives and passivation.

## 4 Experimental Section

### Materials

Lead iodide (PbI_2_, 99.5%), Phenethylamine (PEAI, 99.5%), Methylamine iodide (MAI, 99.5%), and PC_61_BM were purchased from the Xi'an Polymer Light Technology Corp. *N*,*N*‐dimethylformamide (DMF, 99.9%) and carbohydrazide (CBH, 97%) was purchased from Aladdin. All reagents were used without further purification. Corning glass (1.5 cm×1.5 cm) and indium tin oxide (ITO) glass were bought from HT technology.

### Substrate Preparation

Substrates were sequentially ultrasonic‐cleaned with glass cleaning liquid, deionized water, acetone, and ethanol for 20 min, and finally dried with N_2_.

### (PEA)_2_MA_3_Pb_4_I_13_ Precursor Solution Preparation

Phenethylammonium iodide (PEAI), methylammonium iodide (MAI), lead iodide (PbI_2_), and carbohydrazide (CBH)were dissolved in DMF solvent with a molar ratio of 2:3:4:x, where the Pb^2+^ concentration was 0.4 m in the precursor solution and x = 0, 0.5%, 1%, 1.5%, 2%, respectively.

### Device Fabrication

The patterned ITO substrates were treated with air‐plasma for 10 min and immediately transferred into a N_2_‐filled glovebox. The cleaned substrates were preheated at 110 °C for 10 min,^[^ ^4^, ^24^ ^]^ followed by a spin‐coating process of 5000 rpm for 30s with 60 µL of quasi‐2D perovskite solution. The samples were annealed at 100 °C for 10 min and cooled to room temperature. Then 40 µL PC_61_BM solution (20 mg mL^−1^ in chlorobenzene) was coated onto the perovskite film at 2000 rpm for 40s. Finally, 100 nm Ag layer was thermally evaporated as the top electrode with a shadow mask.

### Materials and Devices Characterization

Optical images, surface roughness and thickness of thin films were characterized using laser confocal microscopy (OLS5000). XRD was performed at a scanning rate of 20° min^−1^ with an Empyrean X‐ray diffractometer operated at 40 kV and 30 mA. The UV−vis absorption spectra of the films were collected on a Youke UV‐1901 spectrometer. The Fourier transform infrared (FTIR) absorption spectrum of the films was taken on Nicolet iS50. The steady‐state PL spectrum was measured on an Edinburgh Instruments FLS 980 with an excitation wavelength of 470 nm. Transition absorption (TA) measurements were conducted on a home‐made ultrahigh sensitive transient absorption spectrometer (TAS).^[^ ^17^ ^]^ Scanning electron microscopy (SEM) images were obtained on field emission scanning electron microscopy (FEI inspect) at an acceleration voltage of 8 kV. HRTEM images were obtained on TALOS F200S G2 with an acceleration voltage of 200 kV. The contact angles of deionized water on both films were measured on KRUSS OSA25. *I–V* and *I–t* characteristics of devices were measured on a Keithley 2400 and 6487 digital source meters, respectively. The SCLC curves of the devices were recorded with a keithley 2400 source‐meter. The EIS measurements under dark condition were performed on an CORRTEST CS350M electrochemical workstation. The response time, *EQE*, Responsivity (*R*), Reflectivity (*Ref*.), and transmissivity (*Trans*.) were measured using Enlitech QE‐R3011. And IQE was calculated from the following equation

(1) $$IQE\;=\frac{EQE}{1-Ref.-Trans.}$$

Noise power spectrum was recorded with a home‐made noise measurement system from 0.1 Hz to 100 kHz.

The space charge limited current (SCLC) measurements were performed to characterize the trap density (*N*_d_), which can be determined by the following equation

(2) $$N_{d}=\frac{2\varepsilon_{0}\varepsilon }{eL^{2}}\;V_{\mathrm{TFL}}$$

Where *ε*_0_ is the vacuum dielectric constant, *ε* is the relative dielectric constant, *L* is the thickness of the films, and *V*_TFL_ is the trap‐filling limit voltage.

The responsivity (*R*) indicates the response efficiency of the device to the optical signal and can be defined by the following equation.^[^ ^4^, ^25^ ^]^

(3) $$R\;=\frac{I_{p}-I_{d}}{PS}\;=\frac{EQE\lambda q}{hc}$$

Where *I*_p_, *I*_d_, *S*, and *P* denote the photocurrent, dark current, active area, and light intensity. *𝜆* denotes the wavelength of incident light, *q* is the absolute value of the electronic charge, *h* is the Planck constant, and *c* is the velocity of light in a vacuum.

The detectivity (*D*) is another key parameter that characterizes the ability to detect weak light signals, which can be calculated by the following equation

(4) $$D\;=\frac{R\sqrt{S\cdot B}}{i_{n}}$$

where *B* and *i*_n_ repersent the bandwidth and noise current (Figure S8, Supporting Information).

## Conflict of Interest

The authors declare no conflict of interest.

## Supporting information

Supporting Information

## Data Availability

The data that support the findings of this study are available from the corresponding author upon reasonable request.
