# Correction to “Regulating 3D Phase in Quasi‐2D Perovskite Films for High‐Performance and Stable Photodetectors”

**DOI:** 10.1002/advs.202403805

**Published:** 2024-05-05

*Advanced Science* **2023**, *10*, 2302917.

https://doi.org/10.1002/advs.202302917

The affiliation of authors, W. Zeng and J.‐C. Wang, “University of Chinese Academy of Science, 19A Yuquan Road, Beijing, 100049, China” was incorrect. This should be “University of Chinese Academy of Sciences, 19A Yuquan Road, Beijing, 100049, China.”

We apologize for this error.

