# Supplementary material for: Regulating 3D Phase in Quasi‐2D Perovskite Films for High‐Performance and Stable Photodetectors
Source: Adv Sci (Weinh). 2023 Jul 3;10(26):2302917. doi: 10.1002/advs.202302917 (PMC10502668; doi:10.1002/advs.202302917)
Supplement: Supplementary file 1 — Supporting Information [file ADVS-10-2302917-s001.pdf]

ADVANCED SCIENCE
Open Access

Supporting Information

for *Adv. Sci.*, DOI 10.1002/advs.202302917

Regulating 3D Phase in Quasi-2D Perovskite Films for High-Performance and Stable Photodetectors

Haipeng Di, Wen Zeng, Bo-Han Li, Feiyi Liao, Chen Zhao, Chuanhui Liang, Huang Li, Jia-Cheng Wang, Da-Bing Cheng, Zefeng Ren and Yiying Zhao**

Regulating 3D Phase in Quasi-2D Perovskite films for High-performance and Stable Photodetectors

Haipeng Di [a, †], Wen Zeng [b, c, †], Bo-Han Li [b], Feiyi Liao [a], Chen Zhao [a], Chuanhui Liang [a], Huang Li [b], Jia-Cheng Wang [b, c], Da-Bing Cheng [b], Zefeng Ren [b, *], Yiying Zhao [a, *]

a) Institute of Materials, China Academy of Engineering Physics, Jiangyou 621908, China

b) State Key Laboratory of Molecular Reaction Dynamics, Dalian Institute of Chemical Physics, Chinese Academy of Sciences, Dalian 116023, China

c) University of Chinese Academy of Science, 19A Yuquan Road, Beijing 100049, China.

† These authors contributed equally to this work.

* Authors to whom correspondence should be addressed.

Email addresses: zhaoyiying@caep.cn; zfren@dicp.ac.cn

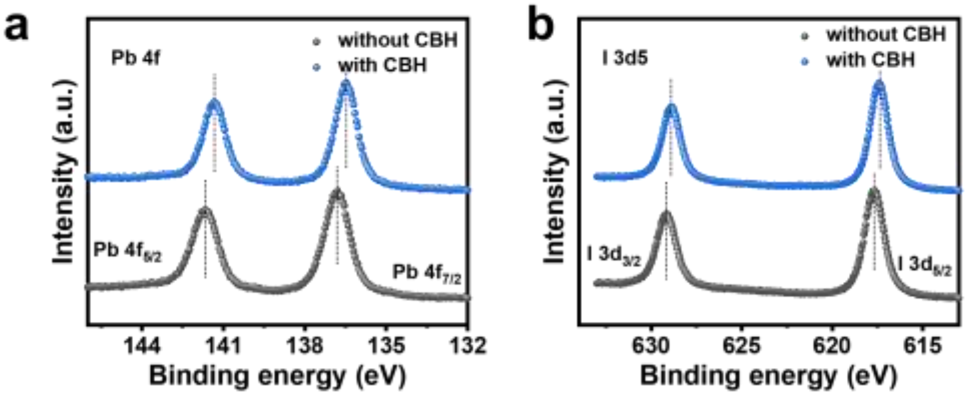

Figure S1 (a) Pb 4f, and (b) I 3d core level in the XPS spectra of the films without and with CBH additive, respectively

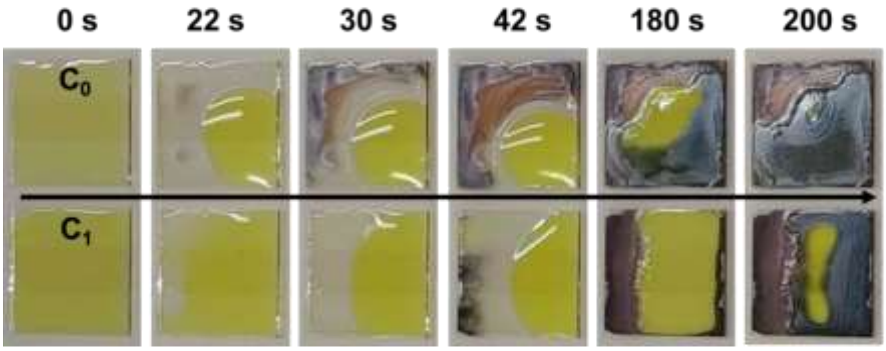

Figure S2 Photographs at different durations of the 30 μL C_0 and C_1 perovskite precursors dropped on the ITO substrates on the hot plate of 70 ^oC

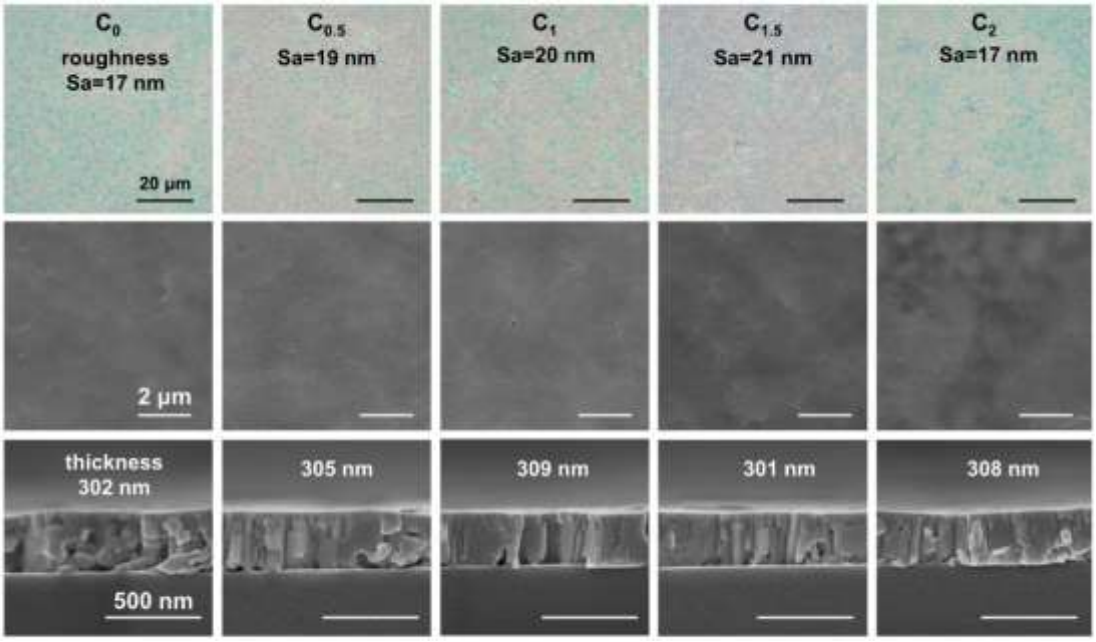

Figure S3 The morphologies of the C_0-C_2 films. (a) Confocal microscopy images, (b) top-view and (c) Cross-sectional SEM images of the C_0-C_2 films, respectively.

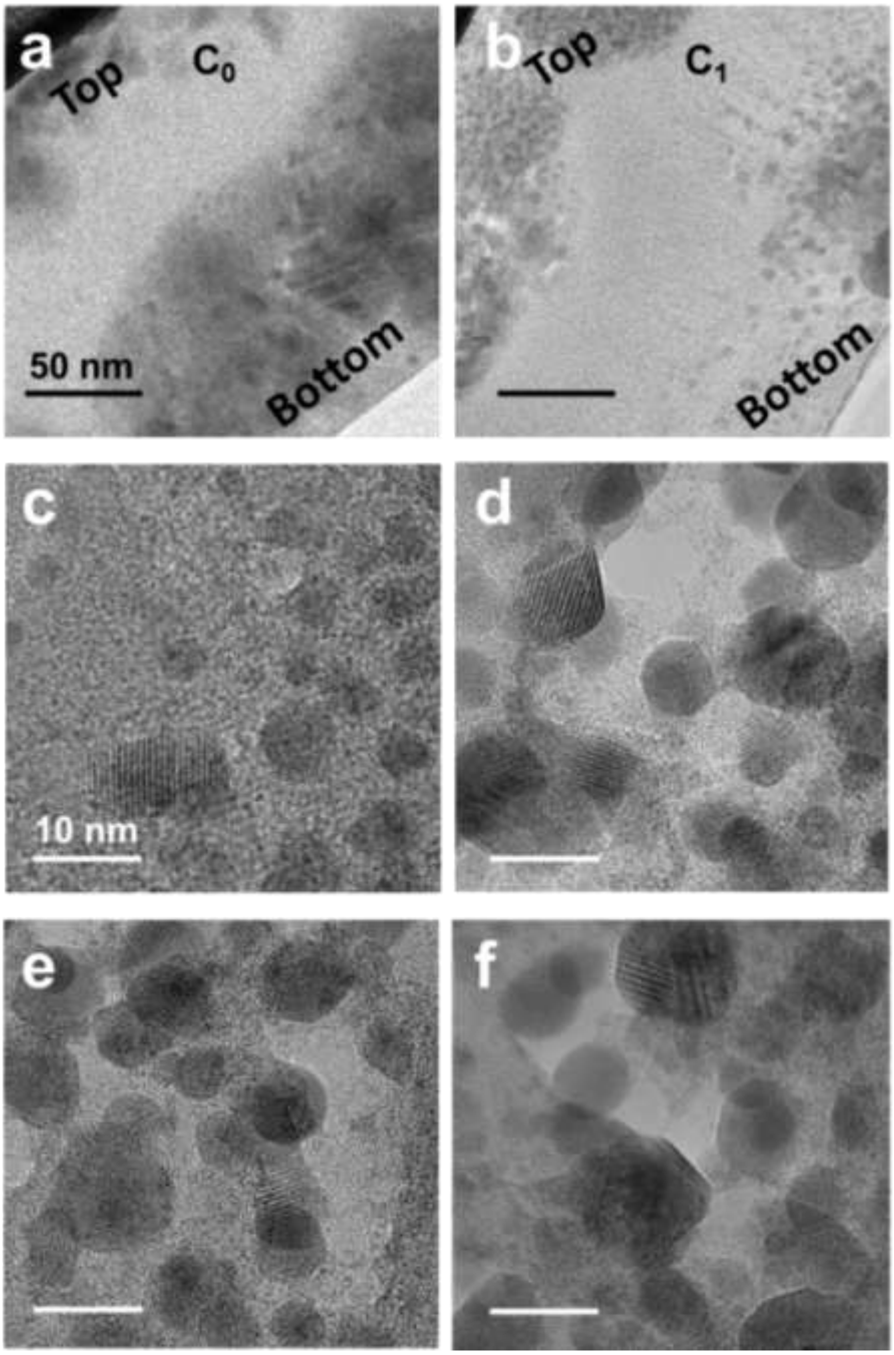

Figure S4 The cross-sectional brightfield TEM image of (a) C_0 and (b) C_1 films. HRTEM images of 3D-perovskite phase in the (c) (e) C_0 and (d) (f) C_1 films, respectively.

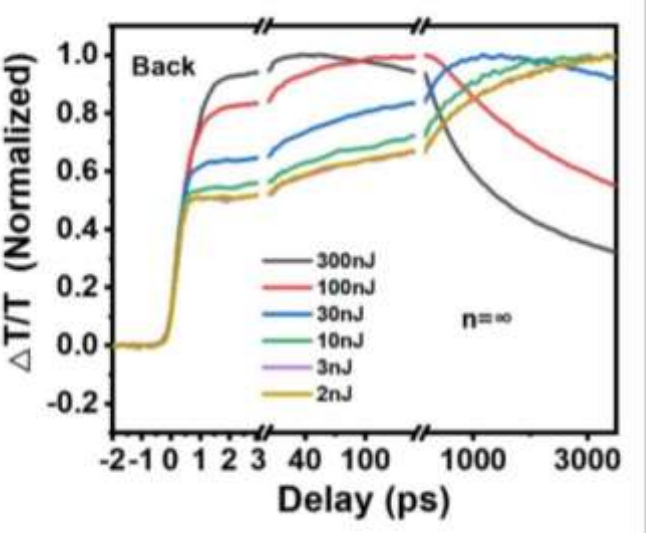

Figure S5 Pump intensity-dependent carrier dynamics. Pump intensity-dependent TA dynamics with a pump wavelength of 517.5 nm and probe at 750 nm for *n*=∞ phases. The normalized ΔT/T were plotted with different pump energies from 300 nJ to 2 nJ per pulse with light spot of about dia. 3 mm. Note the shift in time scale from linear to logarithmic, corresponding to the scale break.

Fitting of dynamic curves of 3D (*n*=∞) phase pumped at 517.5 nm

The bleaching signal of 3D (*n*=∞) phase consists of three ultrafast processes: the direct excitation by pump light, the carrier transfer from the 2D (small-*n*) phases, and the slow decay due to the trap capture and extraction by $PC_{61}BM$ in our work.

The carrier density of 2D phases can be described as

$$\frac{dn_{2D}}{dt} = N_{2D}\delta(t - t_0) - k_T n_{2D} \#(1)$$

Where n_{2D} is the carrier density of 2D phases, and N_{2D} is the total carrier density of the 2D phases generated by photoexcitation, k_T is transfer rate from 2D phases to 3D phase. $\delta(t)$ is defined as

$$\delta(t) = 0, t \neq 0$$

$$\int_{-\infty}^{+\infty} \delta(t)dt = 1 \#(2)$$

By solving Equation 1:

$$\frac{dn_{2D}}{dt} + k_T n_{2D} = N_{2D}\delta(t - t_0)$$

$$e^{k_T t}\left(\frac{dn_{2D}}{dt} + k_T n_{2D}\right) = e^{k_T t} N_{2D}\delta(t - t_0)$$

$$\frac{d}{dt}(n_{2D}e^{k_T t}) = e^{k_T t}N_{2D}\delta(t - t_0)$$

$$\int_{-\infty}^{t}\frac{d}{dt}(n_{2D}e^{k_T t})\,dt = \int_{-\infty}^{t}e^{k_T t}N_{2D}\delta(t - t_0)dt$$

When $t < t_0$, the solution is 0; when $t > t_0$

$$n_{2D}e^{k_T(t)} = e^{k_T t_0}N_{2D}$$

the carrier density of 2D phases can be described by

$$n_{2D}(t) = N_{2D}H(t - t_0)e^{-k_T(t-t_0)}\#(3)$$

H(t) is defined as:

$$H(t) = \begin{cases}1, t > 0\\ 0, t < 0\end{cases} \quad (4)$$

The carrier density of 3D phase can be described as

$$\frac{dn_{3D}}{dt} = N_{3D}\delta(t - t_0) + k_T n_{2D} - Kn_{3D}\#(5)$$

Where n_{3D} is the carrier density of 3D phase, and N_{3D} is the total carrier density of the 3D phase generated by photoexcitation, K is trap capture rate or extraction rate by $PC_{61}BM$.

By solving Equation 5:

$$\frac{dn_{3D}}{dt} = N_{3D}\delta(t - t_0) + k_T\left(N_{2D}H(t - t_0)e^{-k_T(t-t_0)}\right) - Kn_{3D}$$

$$\frac{dn_{3D}}{dt} + Kn_{3D} = N_{3D}\delta(t - t_0) + k_T\left(N_{2D}H(t - t_0)e^{-k_T(t-t_0)}\right)$$

We can divide above Equation to three sections$f_1(t)$, $f_2(t)$ and $f_3(t)$, respectively. and then solve them separately.

$$\begin{cases}\frac{dn_{3D}}{dt} + Kn_{3D} = 0\#f_1(t)\\ \frac{dn_{3D}}{dt} + Kn_{3D} = N_{3D}\delta(t - t_0)\#f_2(t)\\ \frac{dn_{3D}}{dt} + Kn_{3D} = k_T\left(N_{2D}H(t - t_0)e^{-k_T(t-t_0)}\right) \quad \#f_3(t)\end{cases}$$

For $f_1(t)$, the solution is 0.

For $f_2(t)$, the solution is the same as Equation 3 and is described as

$$n_{3D} = N_{3D}H(t - t_0)e^{-K(t-t_0)}\#(6)$$

For $f_3(t)$, the solution is as following:

$$e^{Kt}\left(\frac{dn_{3D}}{dt}+Kn_{3D}\right)=e^{Kt}k_T\left(N_{2D}H(t-t_0)e^{-k_T(t-t_0)}\right)$$

$$\frac{d}{dt}(n_{3D}e^{Kt})=N_{2D}k_Te^{k_Tt_0}e^{(K-k_T)t}\,H(t-t_0)$$

$$\int_{-\infty}^{t}\frac{d}{dt}(n_{3D}e^{Kt})dt=N_{2D}k_Te^{k_Tt_0}H(t-t_0)\int_{-\infty}^{t}e^{(K-k_T)t}dt$$

$$n_{3D}e^{Kt}=\frac{N_{2D}k_Te^{k_Tt_0}}{K-k_T}H(t-t_0)\left(e^{(K-k_T)t}-e^{(K-k_T)t_0}\right)$$

$$n_{3D}=\frac{N_{2D}k_Te^{k_Tt_0}}{K-k_T}H(t-t_0)\left(e^{-k_Tt}-e^{(K-k_T)t_0-Kt}\right)$$

$$n_{3D}=\frac{N_{2D}k_Te^{-k_T(t-t_0)}}{K-k_T}H(t-t_0)\left(1-e^{-(K-k_T)(t-t_0)}\right)\#(7)$$

Combined with Equation 6 and 7, the carrier density of 3D phase can be calculated

$$n_{3D}(t)=\left(N_{3D}e^{-K(t-t_0)}+\frac{N_{2D}k_Te^{-k_T(t-t_0)}}{K-k_T}\left(1-e^{-(K-k_T)(t-t_0)}\right)\right)H(t-t_0)\#(8)$$

The dynamic curves of the n=∞ phase before and after $PC_{61}BM$ coating were fitting with Equation 8 and fitting results are shown below:

Table S1 The detailed fitting parameters of TA curves

	k_T (ps^{-1})	K (ps^{-1})
C_0	4.56×10^{-3}	2.243×10^{-5}
C_1	5.74×10^{-3}	1.231×10^{-5}
$C_0/PC_{61}BM$	/	1.273×10^{-4}
$C_1/PC_{61}BM$	/	2.187×10^{-4}

The electron extraction rate of $PC_{61}BM$ is defined as the variation of K before and after spin coating $PC_{61}BM$. The extraction rates of $PC_{61}BM$ in the C_0 and C_1 films are 1.049×10^{-4} and 2.046×10^{-4} ps^{-1}, respectively.

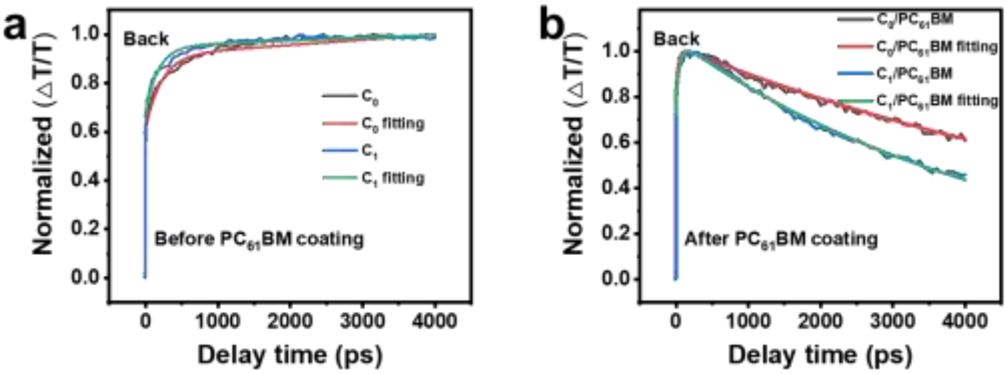

Figure S6 TA dynamic curves and fitting results of the C_0 and C_1 films probed at the 3D (n=∞) bands under back-excitation before $PC_{61}BM$ coating (a) and after $PC_{61}BM$ coating (b).

Table S2 The detailed fitting parameters of TRPL curves

	A_1	τ_1 (ns)	A_2	τ_2 (ns)	A_3	τ_3 (ns)	τ_{ave} (ns)
C_0	4.66%	2.83	15.85%	19.36	79.49%	358.56	288.2
C_1	3.11%	4.49	11.43%	32.92	85.46%	397.63	343.4
$C_0/PC_{61}BM$	5.72%	4.40	17.69%	27.59	76.59%	337.54	263.7
$C_1/PC_{61}BM$	6.43%	2.77	23.71%	20.28	69.86%	290.65	208.0

The TRPL curves are fitted with a three-exponential decay function[1]

$$I = A_1 \exp\left(-\frac{t}{\tau_1}\right) + A_2 \exp\left(-\frac{t}{\tau_2}\right) + A_3 \exp\left(-\frac{t}{\tau_3}\right)$$

Where A_1, A_2 and A_3 are the proportion of three time constant τ_1, τ_2 and τ_3, respectively.

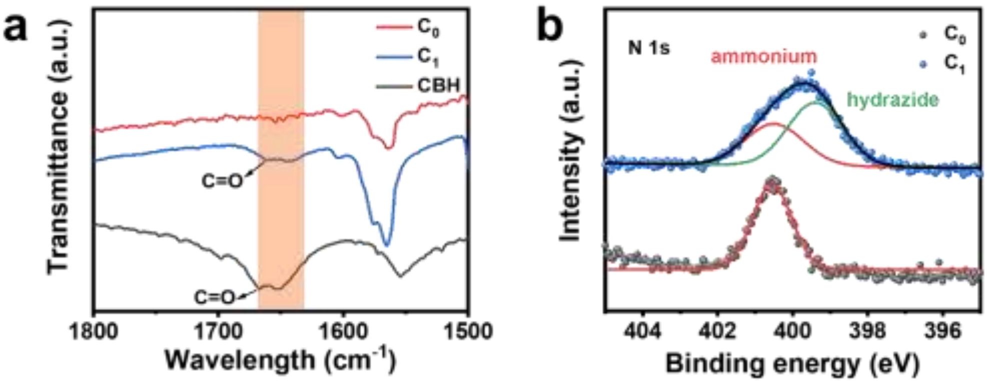

Figure S7 (a) N 1s core level in the XPS spectra of the C_0 and C_1 films, (b) FTIR spectra of neat CBH, C_0, and C_1 films.

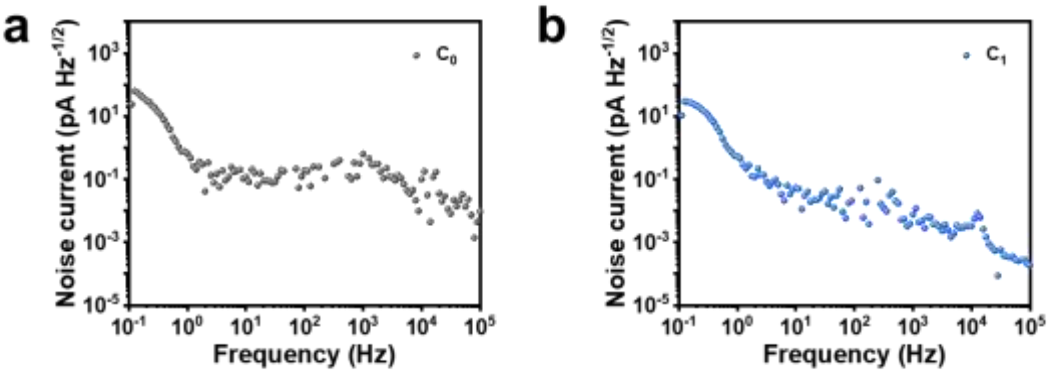

Figure S8 Frequency-dependent noise current of the (a) C_0 and (b) C_1 devices at a bias voltage of 0 V.

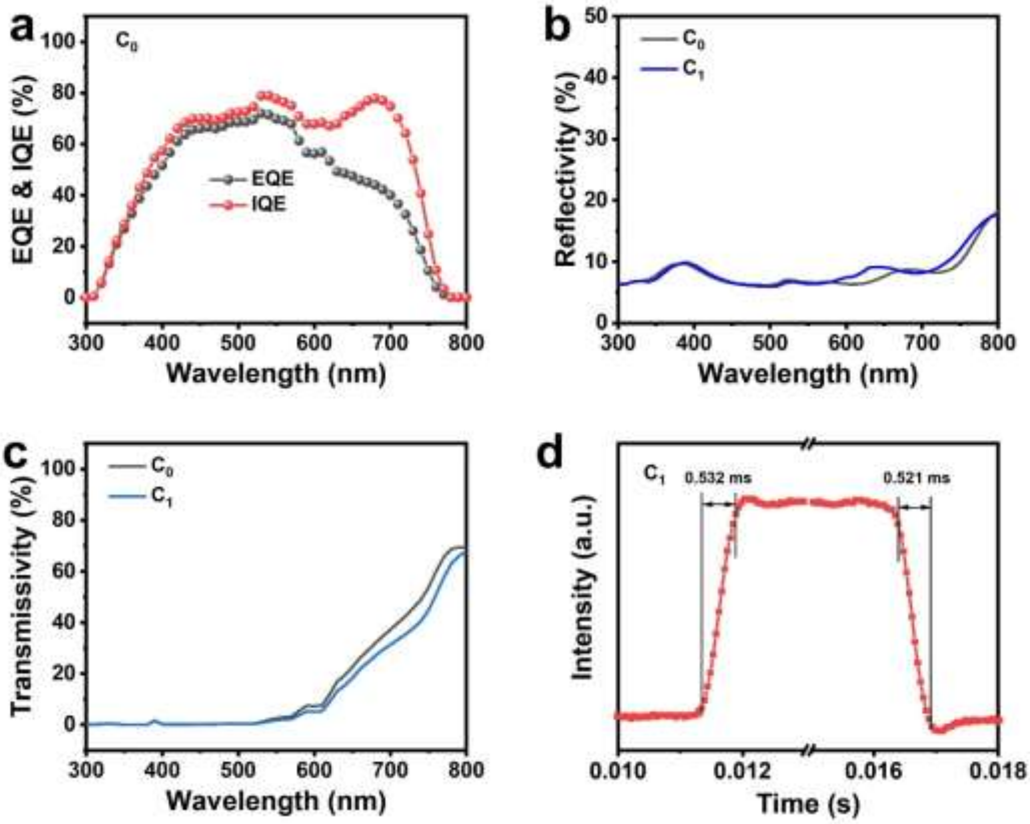

Figure S9 (a) EQE and IQE of the C_0 device. The reflectivity (b) and transmissivity (c) of the C_0 and C_1 films, respectively. (d) Rise and fall times of the C_1 device at 0 V bias.

Table S3 The detailed parameters for comparison with some previously-reported self-powered photodetectors based on 2D/quasi-2D perovskites.

Devices	Bias (V)	Responsivity (*R*) (AW^{-1})	Detectivity (*D*) (Jones)	Ref.
graphene/$(PEA)_2SnI_4/MoS_2$/graphene	0	0.121	8.09×10^9	2
ITO/PEDOT:PSS/$PEA_2MA_3Pb_4I_{13}$/PCBM/Bphen/Al	0	0.46	6×10^{11}	3
ITO/$PEA_2MA_3Pb_4I_{13}$/PCBM/Ag	0	0.44	3.38×10^{12}	4
ITO/PEDOT:PSS/$FPEA_2MA_4Pb_5I_{16}$/PCBM/Ag	0	0.4	/	5
ITO/PEDOT:PSS/$PEA_2MA_4(Sn_{0.5}Pb_{0.5})_5I_{16}$/PCBM/Ag	0	0.38	1.53×10^{12}	6
ITO/PEDOT:PSS/$PEA_2MA_3Pb_4I_{13}/PC_{71}BM$/Ag	0	0.22	1.3×10^{12}	7
ITO/$PEA_2MA_3Pb_4I_{13}$/PCBM/Ag	0	0.41	1.31×10^{12}	This work

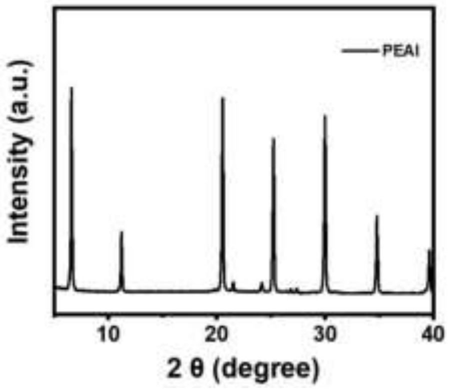

Figure S10 XRD pattern of PEAI.

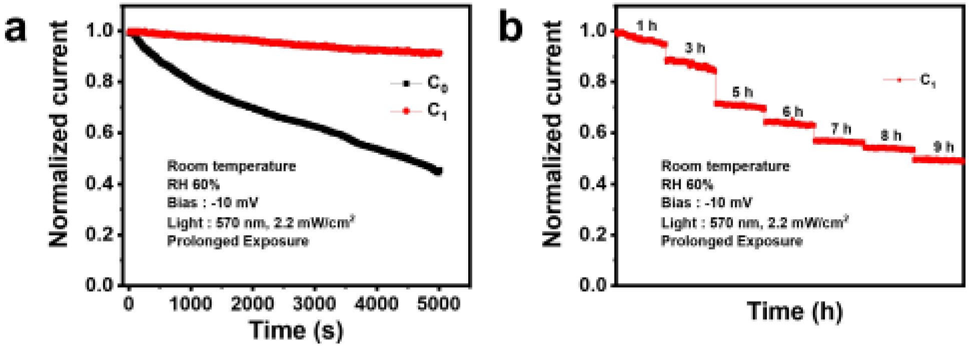

Figure S11 Normalized current variation of (a) C_0 and C_1 devices, (b) C_1 device at a bias voltage of 10 mV with prolonged exposure to light of 570 nm, stored at room temperature with RH 60%.

1 Jiang, W. *et al.* Improving the Performance and High-Field Stability of FAPbBr(3) Single Crystals in X-Ray Detection with Chenodeoxycholic Acid Additive. *Small Methods*, e2201636, doi:10.1002/smtd.202201636 (2023).

2 Fang, C. *et al.* High-Performance Photodetectors Based on Lead-Free 2D Ruddlesden-Popper Perovskite/MoS(2) Heterostructures. *ACS Appl Mater Interfaces* **11**, 8419-8427, doi:10.1021/acsami.8b20538 (2019).

3 Yan, Y. *et al.* Air-Stable and Self-Driven Perovskite Photodiodes with High On/Off Ratio and Swift Photoresponse. *Small* **14**, e1802764, doi:10.1002/smll.201802764 (2018).

4 Min, L., Tian, W., Cao, F., Guo, J. & Li, L. 2D Ruddlesden-Popper Perovskite with Ordered Phase Distribution for High-Performance Self-Powered Photodetectors. *Adv. Mater.* **33**, e2101714, doi:10.1002/adma.202101714 (2021).

5 Zhu, F. *et al.* A General Strategy for Ordered Carrier Transport of Quasi-2D and 3D Perovskite Films for Giant Self-Powered Photoresponse and Ultrahigh Stability. *Nanomicro Lett* **15**, 115, doi:10.1007/s40820-023-01087-5 (2023).

6 Han, Z. *et al.* Oriented Perovskite Growth Regulation Enables Sensitive Broadband Detection and Imaging of Polarized Photons Covering 300-1050 nm. *Adv. Mater.* **33**, e2003852, doi:10.1002/adma.202003852 (2021).

7 Zhang, X., Li, Z., Yan, T., Su, L. & Fang, X. Phase-Modulated Multidimensional Perovskites for High-Sensitivity Self-Powered UV Photodetectors. *Small*, e2206310, doi:10.1002/smll.202206310 (2023).
